# MiR-320b and miR-320d as Biomarkers to Predict and Participate in the Formation of Platinum Resistance in Ovarian Cancer Patients

**DOI:** 10.3389/fonc.2022.881496

**Published:** 2022-05-03

**Authors:** Yuan-Yuan Liu, Ren-Feng Zhao, Chao Liu, Jie Zhou, Liu Yang, Li Li

**Affiliations:** ^1^ Department of Gynecologic Oncology, Guangxi Medical University Cancer Hospital, Key Laboratory of Early Prevention and Treatment for Regional High Frequency Tumor, Ministry of Education, Nanning, China; ^2^ Department of Gynecology, The People’s Hospital of Guangxi Zhuang Autonomous Region, Nanning, China

**Keywords:** platinum-resistant, biomarker, ceRNA network, microRNA, ovarian cancer

## Abstract

Patients with ovarian cancer who receive platinum-based chemotherapy typically develop platinum resistance, which leads to tumor recurrence and mortality. Therefore, finding the underlying mechanisms and biomarkers is critical. A total of 51 platinum-resistant and 70 platinum-sensitive ovarian cancer patients were enrolled in this study. We examined the GSE131978 dataset in the National Center for Biotechnology Information (NCBI) Gene Expression Omnibus database for differentially expressed long non-coding RNAs and messenger RNAs (mRNAs) between platinum-resistant and platinum-sensitive patients and completed a microRNA chip analysis. After filtering by Pearson correlation analysis, the competitive endogenous RNA (ceRNA) networks were subsequently constructed. Then, the Kyoto Encyclopedia of Genes and Genomes (KEGG) and Gene Ontology enrichment analyses about mRNAs in ceRNA networks were accomplished. More crucially, we demonstrated the differentially expressed microRNAs using quantitative real-time PCR and fluorescence *in situ* hybridization. The feasibility of microRNAs as biomarkers to predict platinum resistance and tumor recurrence was assessed using the receiver operating characteristic curve and survival analysis. MiR-320b and miR-320d exhibited high area under the curve values of 0.757 and 0.702, respectively. In our study, ceRNA networks including miR-320b and miR-320d probably provided novel insights for platinum resistance in ovarian cancer patients.

## Introduction

Ovarian cancer is the second most common gynecological cancer in women, with higher recurrence and mortality rates than other cancers (1). Debulking surgery and platinum-based chemotherapy continue to play a key role in the treatment of ovarian cancer, despite the remarkable progress such as precisely targeted medicines and the systemic therapy theory (2). A previous report suggested that the appearance of platinum resistance in chemotherapy is the major cause of the recurrence of advanced ovarian cancer, which eventually leads to death (3). CA125, a widely used clinical biomarker, is recommended for monitoring the response to first-line chemotherapy in ovarian cancer; however, no evidence proves its usefulness (4). Therefore, there is a pressing need to explore the potential molecular mechanisms of platinum resistance in ovarian cancer patients and find novel biomarkers to predict the response of ovarian cancer patients to platinum-based chemotherapy.

Non-coding RNAs (ncRNAs), containing all RNA species except mRNAs, cannot encode proteins or peptides, which can not only directly bind to mRNAs, genes, or nascent transcripts but also indirectly combine histone modifiers to regulate gene expression (5). As competitive endogenous RNAs (ceRNAs), long non-coding RNAs (lncRNAs) competitively combine microRNA response elements (MREs) to modulate the expression of downstream target genes (6). Furthermore, lncRNA can also promote the translation of target genes by enriching RNA-binding proteins (7). Specifically, microRNAs are typically 19–25 nucleotides in size, and recognize the 3’ untranslated region of mRNA to mediate the post-transcriptional silencing of target genes (8). Furthermore, rather than inhibiting gene expression in the cytoplasm, microRNAs can target enhancers to active gene expression in the nucleus (9). Based on these mechanisms, the ceRNA network, constructed by lncRNA–microRNA–mRNA axes, plays a significant role in tumor origination and development, providing novel insights for tumor therapeutic targets and diagnostic biomarkers (10). Nowadays, what has been universally recognized is that quite a lot of microRNAs are significantly upregulated or downregulated in cancer tissues, engaged in multiple cancer pathogenesis and chemotherapy resistance (11, 12). In addition, microRNAs are considered as potential biomarkers for cancer diagnosis and classification, as well as the evaluation of chemotherapeutic drug responsiveness (13).

During the recent decade, there are gradually increasing studies showing that microRNAs specifically target mRNAs to participate in the formation of platinum resistance and immune escape in ovarian cancer (14–16). For instance, the overexpression of miR-30b can induce platinum resistance by inhibiting the expression of MYPT1 (17), whereas miR-211 has been confirmed to improve the platinum sensitivity of ovarian cancer cells by inhibiting the expression of the multiple genes involved in the DNA damage response (18). Moreover, microRNAs serve as efficient biomarkers to predict the response of ovarian cancer patients to platinum, becoming gradually a promising approach to early diagnosis for platinum resistance (19). Further research has shown that circulating microRNAs can effectively predict the response to chemotherapy and the prognosis of ovarian cancer patients (20).

Despite the fact that the foregoing progresses have been achieved, there are still many potential mechanisms to investigate, particularly the lack of effective biomarkers in clinics to predict the response to platinum-based chemotherapy. In the present study, the ceRNA network was constructed using microarray data of ovarian cancer patients with platinum resistance and platinum sensitivity, which was utilized to discover several possible mechanisms and provide novel insights into future research. More importantly, clinical data and tissue samples were timely collected to verify the distinct expression of miR-320b and miR-320d, as well as to evaluate their sensitivities and specificities as biomarkers. This research provides novel insights that miR-320b and miR-320d as biomarkers can predict the response to platinum-based chemotherapy for better therapy regimens in ovarian cancer patients, as well as probably participate in the regulatory mechanisms of the development of platinum resistance.

## Materials and Methods

### Clinical Data Collection

Between January 2011 and August 2021, tissue samples and clinical data were collected retrospectively from 180 patients who received first-line chemotherapy after a debulking surgery at the People’s Hospital of Guangxi Zhuang Autonomous Region. The research was approved by the Ethics Committee of the people’s Hospital of Guangxi Zhuang Autonomous Region and with the written informed consent of all patients. The pathological confirmation of ovarian cancer was obtained from all tissue samples, which were then processed into paraffin sections. The platinum-free interval (PFI) was calculated as the time between the last platinum dose and tumor recurrence, which was higher than 12 months in platinum-sensitive groups and less than 6 months in platinum-resistant groups (21). According to the criteria, 51 platinum-resistant patients and 70 platinum-sensitive patients were eventually enrolled.

### Database Microarray Data Acquisition and Preprocessing

The NCBI Gene Expression Omnibus (GEO, https://www.ncbi.nlm.nih.gov/geo), was a public database comprised of substantial microarray and sequencing data. Based on the GPL570 platform, ovarian tumor tissues from 7 platinum-resistant ovarian cancer patients and 5 platinum-sensitive ovarian cancer patients were selected from the gene expression profile of GSE131978, which was searched using the keywords “platinum-resistant” and “ovarian cancer” (22). Moreover, the microarray rawdata was processed by oligo package (version 1.54.1), including background correction, normalization, and expression computation (23). In order to identify mRNAs and lncRNAs from the probes in the chip, the gene symbol annotation and long non-coding RNA gene annotation (lncRNA) files from GPL570 and Genecode (https://www.gencodegenes.org/) were downloaded, respectively, to further analyze. When more than one probe was annotated to the same gene, the probe with the highest value was chosen to correspond to the target gene.

### MicroRNA Chip Microarray Acquisition and Preprocessing

To complete the microRNA chip analysis, total RNA was extracted from eight ovarian tumor samples, using the Agilent Human miRNA Microarray Kit (DesignID:070156), four of which were platinum resistant and four of which were platinum sensitive. The NanoDrop ND-2000 (Thermo Scientific, Waltham, MA, USA) was used to measure total RNA, while the RNA integrity was evaluated by the Agilent Bioanalyzer 2100 (Agilent Technologies, Palo Alto, CA, USA). The standard chip-processing procedure was implemented, which included sample labeling, microarray hybridization, and washing. Total RNA was first dephosphorylated before being tagged with Cyanine-3-CTP. The tagged RNAs were then hybridized onto the microarray after purification. The arrays were then scanned using an Agilent Scanner G2505C (Agilent Technologies) to get raw pictures, and raw data were extracted using Feature Extraction software (version 10.7.1.1; Agilent Technologies). In the end, the raw data were quantile normalized and filtered by at least 75% detached probes in each sample.

### Identification of Differentially Expressed Long Non-Coding RNAs, MicroRNAs, and mRNAs

The Limma package (version 3.46.0) was used to identify the differentially expressed lncRNAs and mRNAs between platinum-resistant and platinum-sensitive groups. The selection criteria for lncRNAs and mRNAs were both |fold change|>1.5 and p-value <0.05 (24). Meanwhile, the differentially expressed microRNAs were identified with both |fold change|>2 and p-value <0.05 by using Student’s t-test. Subsequently, the results were saved to local Comma—Separated Values (CSV) files for further analysis.

### Prediction of the MicroRNA Targets of Long Non-Coding RNAs and mRNAs

Through starBase and miRWalk, the differentially expressed microRNAs were used to explore interactions with lncRNAs and mRNAs, respectively. The starBase (http://starbase.sysu.edu.cn/index.php), a publicly powerful database to predict relationships between microRNAs and the other RNA, was used to acquire microRNA–mRNA interactions through Ago CLIP-seq Data (25). The miRWalk prediction tool (http://mirwalk.umm.uni-heidelberg.de), a machine learning prediction approach for microRNAs based on the TarPMiR algorithm, was used to obtain data on the microRNAs targeted to mRNAs (26). To increase the accuracy of prediction by microRNAs, the common lncRNAs and mRNAs were recognized as the target genes of microRNAs in the results of the above microarray data and online tools.

### Construction of Competing Endogenous RNA Network

According to the theory of the ceRNA network, the lncRNA–microRNA–mRNA network was constructed as the following approaches: (1) to further improve the accuracy of predicted results, the Pearson correlation coefficient was calculated by using the expression values of the above common lncRNAs and mRNAs; the setting cut-off threshold of lncRNA–mRNA pairs was defined as the coefficient >0.75 and p-value <0.05. (2) Among the above determined lncRNA–mRNA pairs, if the lncRNAs and mRNAs were not only targeted but also identified as negatively co-expressed with the same microRNAs, this competing relationship of the lncRNA–microRNA–mRNA axes would be recognized as the desired result. (3) Then, multiple axes were visualized by utilizing Cytoscape software (version 3.8.2) to construct the ceRNA network.

### Function Enrichment Analysis

The results of Gene Ontology (GO), which included Biological Processes (BP), Cellular Component (CC), and Molecular Function (MF), were obtained using the clusterProfiler software (version 3.18.1) (27). Meanwhile, the KEGG pathway enrichment analysis was also performed through the same method. Both of their thresholds were set as p-value <0.05. At the same time, their results were visualized by utilizing the ggplot2 package (version 3.3.3).

### Real-Time Polymerase Chain Reaction

Total RNA was extracted from 51 platinum-resistant and 70 platinum-sensitive ovarian cancer samples using miRNA extraction Kit DP502 (TIANGEN BIOTECH, Beijing, China), respectively, then reverse transcription was performed through Mir-X miRNA First-Strand Synthesis Kit (TaKaR, Kyoto, Japan). Amplification was completed immediately utilizing TB Green Premix Ex Taq TM II RR820A (TaKaR, Japan). Then, the expressions of interesting microRNAs were detected by QuantStudioTM5 real-time PCR instrument (Applied Biosystems, Foster, CA, USA). Meanwhile, the relative levels of microRNAs and U6 were calculated through the 2^-△△Ct^ method.

### Fluorescence *In Situ* Hybridization Experiment

The platinum-resistant and platinum-sensitive samples of ovarian cancer were processed by the standard fluorescence *in situ* hybridization (FISH) protocol, including Cy3-labeled microRNAs probes and cell nucleus location with 4′,6-diamidino-2-phenylindole (DAPI). The specific probe sequences were as follows: the sequence of miR-320b was 5-CY3-UUGCCCUCUCAACCCAGCUUUU-3, and the sequence of miR-320d was 5-CY3-UCCUCUCAACCCAGCUUUU-3. Three positive fields in each slice were measured for Integrated Optical Density (IOD) and corresponding positive pixel area (Area), and the average optical density (AOD) = IOD/Area was calculated by Image-Pro Plus 6.0 as the criteria for assessing the microRNA difference between platinum-resistant and platinum-sensitive ovarian cancer tissues.

### Receiver Operating Characteristic Curve

The gold standard was utilized to classify platinum-resistant and platinum-sensitive ovarian cancer patients according to the above method. The evaluation criteria of biomarkers were the log2 expression levels of microRNAs measured by RT-PCR in all enrolled patients. The whole data processing including the calculation of the area under the curve (AUC) and the cut-off value was calculated by the pROC package (version 1.18.0). In addition, the sensitivity and specificity of biomarkers were calculated to evaluate the test efficacy.

### Survival Analysis of Redefinition Groups

The cut-off value of microRNAs calculated by the Youden index in the ROC curves was used to define high- and low-expression groups. The patients whose microRNA expression was higher than the cut-off value were assigned to be in the high-expression group, whereas patients in the low-expression group were recognized as having lower microRNA expressions than the cut-off value. The survival curves of the two groups were plotted using the survival package (version 3.2-7) to look for statistical differences in tumor relapse using the log-rank test.

### Statistical Analysis

All data were analyzed and visualized with R version 4.0.4, IBM SPSS Statistics 26.0, and GraphPad Prism 6.0 software. The continuous and categorical variables were analyzed by the Mann–Whitney U test and Pearson chip-squared test, respectively. Both univariate and multivariate Cox analyses were used to identify independent risk factors of progression-free survival (PFS) between platinum-resistant and platinum-sensitive groups. All significant indicators were defined as a p-value <0.05.

## Results

### Clinical Characteristics

The study enrolled 121 patients with ovarian cancer who were treated with platinum-based first-line chemotherapy after surgery. According to the PFI, participants were divided into two groups, including 51 platinum-resistant patients and 70 platinum-sensitive patients, respectively. The clinical characteristics of the two groups were compared, as well as noteworthy discrepancies were enumerated between them (**Table 1**). There was no statistical difference between the two groups in terms of age, the initial tumor location, or distant metastasis; however, ovarian cancer patients with a higher Federation International of Gynecology and Obstetrics (FIGO) stage and more metastatic sites were considered to be more prone to platinum resistance. Furthermore, risk factors in patients were discovered using univariate and multivariate Cox analyses. FIGO stages III–IV, appendix metastasis positive, and lymph node metastasis positive were all identified as adverse independent risk factors for PFS in 121 patients, with hazard ratios of 6.656, 2.752, and 2.263, respectively (**Table 2**).

**Table 1:** Clinical characteristics between platinum-resistant and platinum-sensitive groups.

	Number	Platinum resistant	Platinum sensitive	P-value
Age (year)*	54.0 (49.0-62.0)	55.0 (49.0-62.0)	54.0 (49.0-61.0)	0.821
**FIGO Stage**				0.000
I–II	40 (33.1%)	5 (4.2%)	35 (28.9%)	
III–IV	81 (66.9%)	46 (38.0%)	35 (28.9%)	
**Primary tumor location**				0.146
Unilateral	68 (56.7%)	25 (20.8%)	43 (35.8%)	
Bilateral	52 (43.3%)	26 (21.7%)	26 (21.7%)	
**Residual carcinoma diameter**				0.006
<1 cm	99 (81.8%)	36 (29.8%)	63 (52.1%)	
>1 cm	22 (18.2%)	15 (12.4%)	7 (5.8%)	
**Metastatic sites**				
**Pelvic and abdominal metastases**				0.000
Positive	74 (61.2%)	43 (35.5%)	31 (25.6%)	
Negative	47 (38.8%)	8 (6.6%)	39 (32.2%)	
**Appendix metastasis**				0.000
Positive	48 (39.7%)	34 (28.1%)	14 (11.6%)	
Negative	73 (60.3%)	17 (14.0%)	56 (46.3%)	
**Greater omentum metastasis**				0.000
Positive	63 (52.1%)	42 (34.7%)	21 (17.4%)	
Negative	58 (47.9%)	9 (7.4%)	49 (40.5%)	
**Intestinal metastasis**				0.001
Positive	67 (55.4%)	37 (30.6%)	30 (24.8%)	
Negative	54 (44.6%)	14 (11.6%)	40 (33.1%)	
**Lymph node metastasis**				0.000
Positive	46 (38.0%)	32 (26.4%)	14 (11.6%)	
Negative	75 (62.0%)	19 (15.7%)	56 (46.3%)	
**Distant metastasis**				0.075
Positive	22 (18.2%)	13 (10.7%)	9 (7.4%)	
Negative	99 (81.8%)	38 (31.4%)	61 (50.4%)	
**Ascites cytology**				0.000
Positive	78 (64.5%)	44 (36.4%)	34 (28.1%)	
Negative	43 (35.5%)	7 (5.8%)	36 (29.8%)	

*Median (first quartile–third quartile).

**Table 2:** Univariate and multivariate Cox proportional hazards regression analysis of risk factors associating with PFS in 121 patients.

	Univariate Cox regression analysis				Multivariate Cox regression analysis			
	Coefficient	HR	95%CI	P-value	Coefficient	HR	95%CI	P-value
**Age**	0.012	1.012	0.973-1.054	0.544				
**FIGO stage**								
III–IV versus I–II	2.923	18.588	4.304-80.281	0.000	1.896	6.656	1.313-33.737	0.022
**Primary tumor location**								
Bilateral versus unilateral	1.213	3.362	1.678-6.739	0.001				
**Diameter of residual carcinoma**								
>1 cm versus <1 cm	1.634	5.124	2.630-9.983	0.000				
**Pelvic and abdominal metastases**								
Positive versus negative	1.536	4.648	1.998-10.811	0.000				
**Appendix metastasis**								
Positive versus negative	2.030	7.615	3.586-16.171	0.000	1.012	2.752	1.239-6.111	0.013
**Greater omentum metastasis**								
Positive versus negative	2.251	9.945	3.858-23.372	0.000				
**Intestinal metastasis**								
Positive versus negative	1.565	4.781	2.095-10.912	0.000				
**Lymph node metastasis**								
Positive versus negative	1.910	6.750	3.228-14.117	0.000	0.817	2.263	1.032-4.963	0.041
**Distant metastasis**								
Positive versus negative	0.678	1.971	0.925-4.200	0.079				
**Ascites cytology**								
Positive versus negative	1.964	7.124	2.507-20.246	0.000				

HR, hazard ratio; CI, confidence interval.

### Identification of Differentially Expressed lncRNAs, microRNAs, and mRNAs

**Figure 1:**
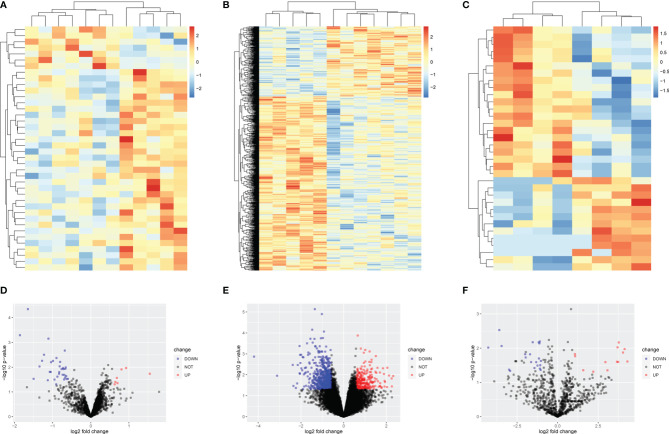
Heatmap and volcano plot of differentially expressed lncRNAs, mRNAs, and microRNAs. **(A)** Heatmap of 7 upregulated and 33 downregulated lncRNAs. **(B)** Heatmap of 243 upregulated and 604 downregulated MRNAs. **(C)** Heatmap of 13 upregulated and 21 downregulated lncRNAs. **(D)** Volcano plot of 7 upregulated and 33 downregulated lncRNAs. **(E)** Volcano plot of 243 upregulated and 604 downregulated mRNAs. **(F)** Volcano plot of 13 upregulated and 21 downregulated microRNAs.

After preprocessing, the general expressions of reading count matrix including GSE131978 and the microRNA chip microarray were visualized using a boxplot (**Supplementary Figures 1A–C**). Then, principal component analysis (PCA), a linear dimensionality reduction algorithm, was utilized to evaluate differences in platinum-resistant and platinum-sensitive patients by variance (**Supplementary Figures 1D–F**). Between platinum-resistant and platinum-sensitive groups, 33 downregulated and 7 upregulated lncRNAs, 604 downregulated and 243 upregulated mRNAs, 21 downregulated microRNAs, and 13 upregulated microRNAs were identified using the above selection criteria (**Supplementary Tables 1–3**). Moreover, the heatmap and volcano plot were used to better visualize the differentially expressed mRNAs (**Figures 1A–F**).

### Construction of lncRNA–microRNA–mRNA Competitive Endogenous RNA Network

**Figure 2:**
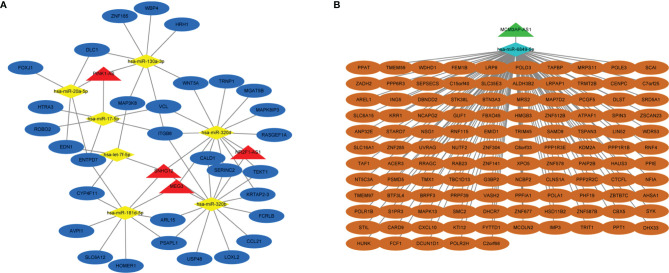
Construction of lncRNA-mediated ceRNA regulatory network. **(A)** The upregulated lncRNA-mediated ceRNA regulatory network. **(B)** The downregulated lncRNA-mediated ceRNA regulatory network. Red means upregulated lncRNAs, yellow means downregulated microRNAs and mRNAs, blue means upregulated lncRNAs and microRNAs, and green means downregulated lncRNAs.

Based on the ceRNA network theory, mRNAs with a Pearson correlation coefficient >0.75 and p-value <0.05 were further identified as hub mRNAs, including 463 downregulated and 68 upregulated mRNAs, which were used to construct the ceRNA network in the following. By merging previously predicted results and microarray data, a total of 214 lncRNA–miRNA–mRNA axes were discovered, containing 5 lncRNAs, 8 microRNAs, and 146 mRNAs. Eventually, two ceRNA networks were visualized: the upregulated lncRNA-mediated network comprised 4 lncRNAs, 7 microRNAs, and 31 mRNAs, and the downregulated lncRNA-mediated network consisted of 1 lncRNA, 1 microRNA, and 115 mRNAs (**Figures 2A, B**). The mRNAs in two ceRNA networks were listed (**Supplementary Table 4**). The top two most connected microRNAs in ceRNA networks were miR-320b and miR-320d, each of which targeted 3 lncRNAs and 11 mRNAs. Even more interestingly, they were targeted at 3 lncRNAs at the same time, in particular SNHG12, MEG3, and NR2F1-AS1, as well as respectively engaged in 33 lncRNA–microRNA–mRNA axes. According to the ceRNA theory, lncRNAs frequently competitively combine MREs to drive target gene posttranscriptional silencing, implying that miR-320b and miR-320d may play an essential role in platinum resistance (**Supplementary Table 5**).

### Function Enrichment Analysis of mRNAs in Competitive Endogenous RNA Network

**Figure 3:**
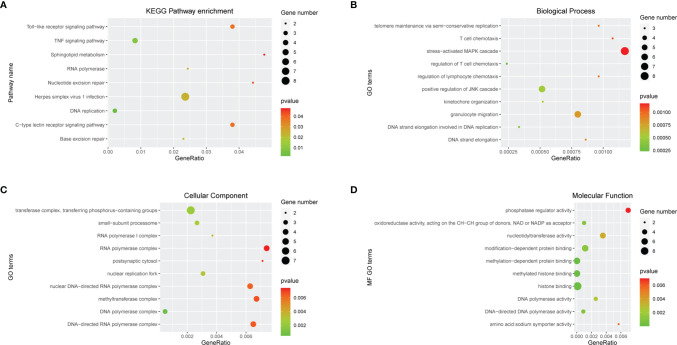
Function enrichment analysis of mRNAs in ceRNA network. **(A)** KEGG enrichment analysis of mRNAs in ceRNA network. **(B-D)** GO enrichment analysis of mRNAs in ceRNA network including BP, CC, and MF. Gene numbers represent the number of enriched mRNAs.

**Figure 4:**
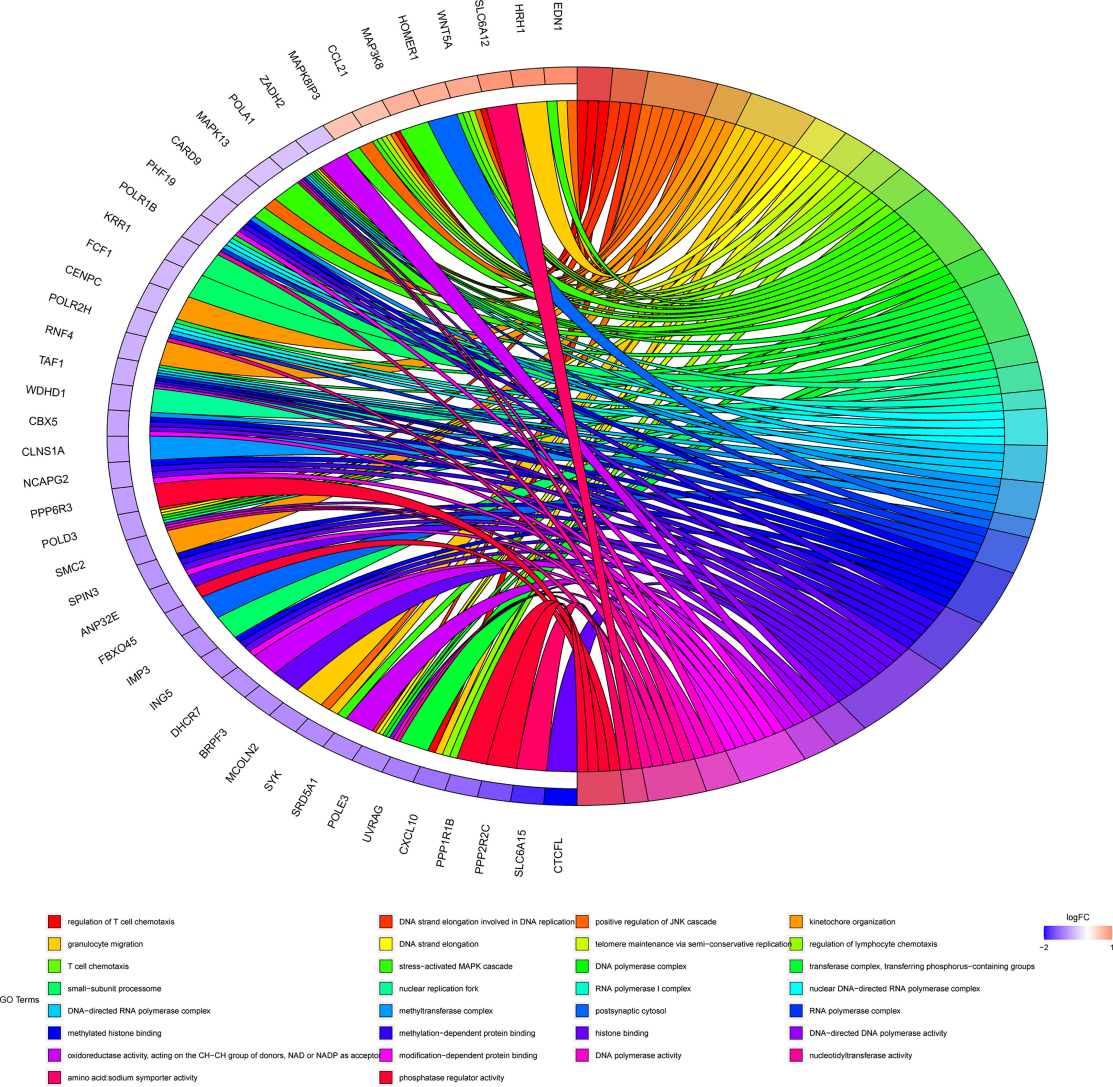
Chord diagram of top 10 BP, CC, and MF. Different colors on the right of the chord diagram represent different GO terms, corresponding to legend, the left of the chord diagram represents mRNAs enriched in different GO terms, and the line in the middle represents mRNAs that are enriched in GO terms.

In order to further clarify the physiological functions of mRNAs in the ceRNA networks, the KEGG pathway and GO function enrichment analysis were utilized to discover potential pathways and functions of platinum-resistant in ovarian cancer patients. The KEGG pathway analysis was primarily focused on “DNA replication”, “Base excision repair”, “Nucleotide excision repair”, and “RNA polymerase”, all of which had great opportunities to be involved in the progression of platinum-resistant cancers, in addition, the mRNAs were also highly relevant to inflammation-associated pathways, such as “TNF signaling pathway”, “Toll-like receptor signaling pathway”, and “C-type lectin receptor signaling pathway” (**Figure 3A**). Meanwhile, we further demonstrated the participation of mRNAs in different KEGG pathways (**Supplementary Figure 2**). GO function enrichment analysis revealed that “telomere maintenance *via* semi-conservative replication,” “DNA strand elongation involved in DNA replication,” and “stress-activated MAPK cascade” were enriched in the biological process and the cellular component consisted of “DNA polymerase complex” and “nuclear replication fork”; meanwhile, “methylated histone binding” and “DNA polymerase activity” participated in molecular function (**Figures 3B–D**). In the end, the top 10 GO terms of BP, CC, and MF, respectively, were visualized to observe the mRNAs involved in enrichment results (**Figure 4**).

### Expression of miR-320b and miR-320b in the Ovarian Samples by Real-Time Polymerase Chain Reaction

**Figure 5:**
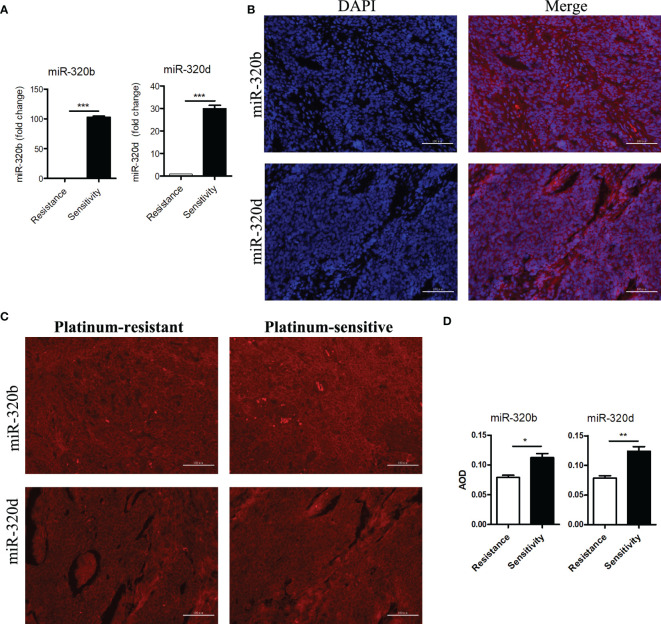
Differential expressions of miR-320b and miR-320d between platinum-resistant and platinum-sensitive ovarian cancer tissues were verified by RT-PCR and FISH. **(A)** Expression levels of miR-320b and miR-320d in ovarian cancer tissues by RT-PCR. **(B)** Cellular localization of miR-320b and miR-320d in ovarian cancer tissues by FISH. **(C, D)** Quantitative analysis of miR-320b and miR-320d in ovarian cancer tissues by FISH. ***p<0.001. RT-PCR, real-time polymerase chain reaction; FISH, fluorescence *in situ* hybridization experiment. *p < 0.05; **P < 0.01.

After evaluating PFI in patients receiving platinum-based first-line chemotherapy, total RNA was extracted from 51 platinum-resistant and 70 platinum-sensitive ovarian tumor tissues. Then, RT-PCR was performed according to the standard protocol. Our findings revealed that the expression levels of miR-320b and miR-320d in the platinum-sensitive group were significantly higher than those in the platinum-resistant group, and the fold changes were 102.9 and 29.9 times, respectively, both with a p-value <0.001 (**Figure 5A**).

### Cytoplasm Localization and Expression of miR-320b and miR-320b in the Ovarian Samples by Fluorescence *In Situ* Hybridization

To further verify the differences of miR-320b and miR-320d expression levels between platinum-resistant and platinum-sensitive groups, FISH was performed by the standard protocol. MiR-320b and miR-320d were observed in the cytoplasm based on the DAPI-labeled blue nucleus, indicating that they probably specifically target the 3 ‘ untranslated region of mRNAs to mediate post-transcriptional silencing (**Figure 5B**). Meanwhile, the average optical density (AOD) of miR-320b and miR-320d in the platinum-sensitive group was higher than the platinum-resistant group with a p-value <0.05 (**Figures 5C, D**).

### Sensitivities and Specificities of miR-320b and miR-320d in the Prediction of Platinum Resistance

**Figure 6:**
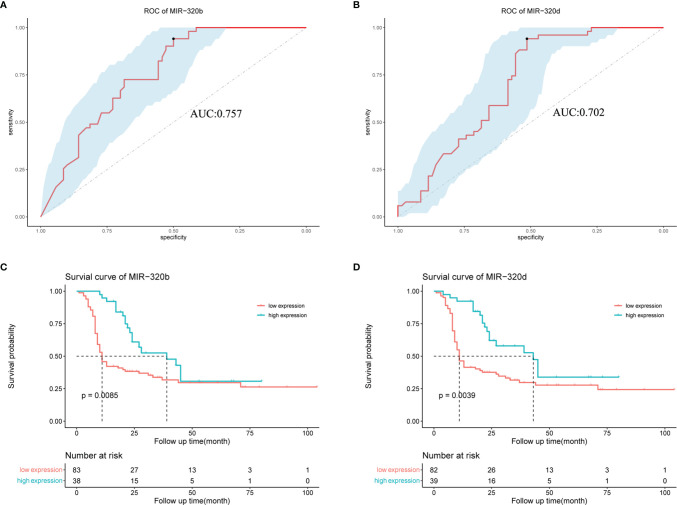
Effect of miR-320b and miR-320d as biomarkers to predict platinum resistance and tumor recurrence in ovarian cancer patients receiving platinum-based chemotherapy by ROC and survival curves. **(A, B)** ROC curves of miR-320b and miR-320d. **(C, D)** Survival curves of high-expression group and low-expression group judged by expression levels of miR-320b and miR-320d, respectively. ROC curve, receiver operating characteristic curve.

MiR-320b and miR-320d were low expressions in platinum-resistant ovarian cancer samples; thus, ROC curves were used to estimate their sensitivities and specificities in order to see if they might be utilized as biomarkers to reliably predict a response to platinum-based chemotherapy (**Figures 6A, B**). According to our findings, miR-320b and miR-320d exhibited higher AUC values of 0.757 and 0.702, respectively. Meanwhile, there were extremely excellent sensitivities in diagnosing platinum resistance, which were both 0.941; the disappointing fact was that the specificities of miR-320b and miR-320d were 0.500 and 0.514, respectively, relatively low. Our findings showed that the miR-320b and miR-320d expression levels measured during surgery approximately predict 94% of platinum-resistant patients and 50% of platinum-sensitive patients, allowing patients to begin more effective treatments earlier and prevent a tumor relapse. More common statistical indicators, such as the predictive value, likelihood ratio, and relative risk, were listed (**Supplementary Table 6**).

### Survival Analysis of High-Expression and Low-Expression Groups

The survival curves were utilized to see if there were any differences in tumor relapse between the high- and low-expression groups of miR-320b and miR-320d, as shown in the figure below. Our findings revealed that ovarian cancer patients with low miR-320b and miR-320d expression had a greater rate of tumor relapse, with p-values of 0.0085 and 0.0039, respectively (**Figures 6C, D**). However, there was no significant difference in overall survival (OS) between two groups, the better of which was defined by miR-320b with a p-value of 0.16 and the other by miR-320d with a p-value of 0.97, which could be related that the therapeutic regimen changed after tumor recurrence and some patients were still alive at the end of the follow-up (**Supplementary Figures 3A, B**). Based on these findings, miR-320b and miR-320d could successfully predict tumor recurrence but not OS in ovarian cancer patients receiving platinum-based chemotherapy.

## Discussion

In this study, we constructed the lncRNA-mediated ceRNA networks to reveal that miR-320b and miR-320d were probably involved in the mechanisms of platinum resistance in ovarian cancer patients mediated by SNHG12, MEG3, and NR2F1-AS1. Moreover, we further demonstrated that they can be used as novel biomarkers to early predict platinum resistance and tumor relapse in ovarian cancer patients during surgery. Specifically speaking, we collected clinical data and samples from 51 platinum-resistant and 70 platinum-sensitive ovarian cancer patients receiving first-line chemotherapy according to the prescribed assessment criteria from Gynecologic Cancer InterGroup (GCIG) (21). Based on the methods described above, two ceRNA networks encompassing 5 lncRNAs, 8 microRNAs, and 146 mRNAs were constructed using the combination of microarray data and GEO database. The differentially expressed mRNAs in two ceRNA networks were predominantly enriched in DNA synthesis and damage repair processes, such as “Base excision repair” and “Nucleotide excision repair,” according to the KEGG pathway and GO function enrichment analysis. Meanwhile, the top two most tightly connected microRNAs in ceRNA networks were miR-320b and miR-320d, which were significantly downregulated in platinum-resistant ovarian cancer samples by RT-PCR and FISH, with p-value <0.05. They were proven in the cytoplasm by FISH and respectively involved in 33 lncRNA–microRNA–mRNA axes, suggesting that they were involved in the post-transcriptional silencing of target genes in the occurrence and progression of platinum-resistant ovarian cancer. In ovarian cancer patients, miR-320b and miR-320d were confirmed as novel biomarkers for predicting the response to platinum-based first-line chemotherapy and tumor relapse, with higher AUC values, 0.757 and 0.702, respectively.

In the upregulated lncRNA-mediated ceRNA network, 3 lncRNAs simultaneously targeted miR-320b and miR-320d, in particular SNHG12, MEG3, and NR2F1-AS1. SNHG12 was involved in unfolded protein responses in many tumor cells, which were exploited to evade the immune-mediated attack and correlated with chemotherapeutic or targeted drug resistance in various malignancies (28). Recent evidence suggested that the overexpression of SNHG12 specifically combined miR-129-5p in the glioblastoma, causing the upregulation of MAPK1 and E2F7, which impaired temozolomide (TMZ)-induced apoptosis and the G1/S cell cycle conversion, resulting in the appearance of acquired TMZ resistance (29). Moreover, the increased expression of SNHG12 promoted the development of doxorubicin resistance through the SNHG12/miR-320a/MCL1 axis in osteosarcoma cells (30). In our study, upregulated SNHG12 may have participated in the underlying mechanisms of platinum resistance in ovarian cancer patients by targeting miR-320b and miR-320d. In colorectal cancer, non-small cell lung cancer, thyroid cancer, and acute myeloid leukemia, MEG3 was found to have a high expression in the treatment-sensitive group and to combine the relevant microRNAs to improve therapy sensitivity (31–34). However, we discovered that MEG3 was upregulated in platinum-resistant ovarian cancer patients, which was most probably due to the difference in the disease type and therapy regimen. Similar to the ceRNA network, NR2F1-AS1 was upregulated in oxaliplatin-resistant hepatocellular carcinoma patients and promoted ABCC1 expression by targeting miR-363 to increase chemoresistance (35). Further research revealed that miR-320b was significantly downregulated and suppressed apoptosis in adriamycin-resistant breast cancer cell lines *via* negatively regulating RBM38, while the overexpression of miR-320b produced the opposite effects (36). Low expression of miR-320b resulted in a weakened inhibition of RAD21 in hepatocellular carcinoma treated with ionizing radiation, resulting in the reduction of therapy-induced DNA damage (37). These results were consistent with our findings that miR-320b played an essential role in therapeutic resistance not only in chemotherapy but also in radiotherapy. Moreover, a recent study showed that miR-320b was downregulated in TMZ-resistant glioblastoma stem-like cells by RNA-seq and RT-PCR (38). Just as we described earlier, lncRNAs generally silenced the mRNA expression by competitively binding microRNAs with the sponge effect, affecting the response to therapy in multiple cancers. Patients with platinum-resistant ovarian cancer have tried a variety of therapeutic regimens, including nonplatinum-based therapy, to prolong PFI and multiple drug combinations (39). The Best Approach in Recurrent-Ovarian-Cancer-with Cediranib-Olaparib (BAROCCO) trial showed that the combination of cediranib–olaparib did not significantly improve PFS in platinum-resistant patients compared with paclitaxel (40). Nonetheless, a few studies indicated that the underlying mechanisms about the initiation and progression of platinum-resistant in ovarian cancer patients. In our study, the lncRNA–microRNA–mRNA axes identified in ceRNA networks may provide novel insights to the mechanisms of platinum resistance.

The KEGG pathway and GO function enrichment analysis were performed on 31 upregulated and 115 downregulated mRNAs in the ceRNA networks to further investigate the physiological roles of differentially expressed mRNAs in ovarian cancer patients with platinum resistance. Our findings revealed that “Base excision repair” and “Nucleotide excision repair” were obviously correlated to the occurrence of platinum resistance, which was consistent with a previous study that aberrant activity in one of the various DNA repair pathways was general in cancers and may enhance their repair capability or tolerate platinum-induced DNA damage, eventually leading to platinum resistance (41). Furthermore, we also noticed these mRNAs were enriched in “methylated histone binding” and “histone binding,” particularly recent research suggested that treatment with a histone deacetylase inhibitor reduced the functions of ACTL6A overexpression, resulting in not only decreasing repair of cisplatin-DNA adducts but also improving sensitivity to cisplatin therapy (42). As a result, it was reasonable to assume that histone modification was crucial in the generation of platinum-resistant cells. Moreover, there were also some enrichment results linked to DNA and RNA syntheses, which may result in platinum resistance.

Multiple studies have reported that platinum-based chemotherapy was still an essential treatment for ovarian cancer, but some patients inevitably experienced tumor relapse and death due to platinum resistance (2, 3). Obviously, it was critical to discover clinical indicators that might accurately predict platinum reactivity. During the last decade, microRNAs have universally participated in the development of drug resistance (43). It provided the basis for microRNAs as biomarkers to predict the medication response. Four microRNAs encompassing miR-22-3p, miR-98-5p, miR-454-3p, and miR-183-5p were evaluated in high-grade serous ovarian cancer with the ability to distinguish platinum-resistant and platinum-sensitive patients and predict their prognosis (44). Moreover, let-7g, which can be extracted from both serum and tissue, has been recommended as a new biomarker for predicting chemotherapy resistance in epithelial ovarian cancer (19). However, no research has been reported to indicate that miR-320b and miR-320d may be used as biomarkers to predict the response to platinum-based chemotherapy. Therefore, we detected the expression levels of miR-320b and miR-320d in 51 platinum-resistant and 70 platinum-sensitive ovarian cancer patients by RT-PCR to evaluate their responses to platinum-based chemotherapy. Our results revealed that miR-320b and miR-320d were effective to determine platinum resistance, with the AOC values of 0.757 and 0.702, respectively. Specifically speaking, they were more suitable for detecting platinum-resistant ovarian cancer patients in clinic, with both having high sensitivities of 0.941. However, low specificities of 0.500 and 0.514 were obtained in detecting platinum-sensitive ovarian cancer patients, respectively. And more interestingly, tumor relapse was adversely linked with the expression levels of miR-320b and miR-320d in ovarian cancer patients who received platinum-based first-line chemotherapy after debulking surgery.

## Conclusions

In this study, the ceRNA networks were constructed through chip data and online prediction results to search for the underlying mechanisms of platinum resistance. The top two most connected microRNAs, miR-320b and miR-320d, have been demonstrated to be downregulated in platinum-resistant tissues. More intriguingly, they are both specifically combined with 3 lncRNAs including SNHG12, MEG3, and NR2F1-AS1, probably providing novel insights into the regulatory mechanisms of platinum resistance. We further divided 51 platinum-resistant and 70 platinum-sensitive ovarian cancer patients into the high-expression group and low-expression group according to expression levels of miR-320b and miR-320d, confirming that they can predict the formation of platinum resistance and tumor recurrence in ovarian cancer patients during surgery. However, the underlying mechanisms predicted by the ceRNA networks containing miR-320b and miR-320d should be further verified in platinum-resistant ovarian cancer cell lines and tumor xenograft models.

## Data Availability Statement

The original contributions presented in the study are included in the article/**Supplementary Material**. Further inquiries can be directed to the corresponding authors.

## Ethics Statement

The studies involving human participants were reviewed and approved by the Ethics Committee of the People’s Hospital of Guangxi Zhuang Autonomous Region. The patients/participants provided their written informed consent to participate in this study.

## Author Contributions

YL and LL contributed to the design of the study and manuscript writing. LL edited the manuscript. RZ, CL, and JZ were responses for data collection. YL contributed to data management. CL and JZ participated in data analysis. All authors have read and approved the published version of the manuscript.

## Conflict of Interest

The authors declare that the research was conducted in the absence of any commercial or financial relationships that could be construed as a potential conflict of interest.

## Publisher’s Note

All claims expressed in this article are solely those of the authors and do not necessarily represent those of their affiliated organizations, or those of the publisher, the editors and the reviewers. Any product that may be evaluated in this article, or claim that may be made by its manufacturer, is not guaranteed or endorsed by the publisher.

## Supplementary Material

Supplementary Figure 1 Boxplot and PCA of differentially expressed lncRNAs, mRNAs and microRNAs. **(A-C)** Boxplot of differentially expressed lncRNAs, mRNAs and microRNAs between platinum-resistant and platinum-sensitive groups, respectively. **(D-F)** PCA of differentially expressed lncRNAs, mRNAs and microRNAs between platinum-resistant and platinum-sensitive groups, respectively. PCA, Principal Component Analysis; PR, platinum-resistant; PS, platinum-sensitive.

Supplementary Figure 2 Chord diagram of mRNAs enriched in KEGG pathways. Different colors on the right of the chord diagram represent different KEGG pathways, corresponding to legend, the left of the chord diagram represents mRNAs enriched in different KEGG pathways, and the line in the middle represents mRNAs are enriched in KEGG pathways.

Supplementary Figure 3 Effect of miR-320b and miR-320d as biomarkers to predict overall survival in ovarian cancer patients receiving platinum-based chemotherapy by survival curves. **(A, B)** Survival curves of high expression group and low expression group judged by expression levels of miR-320b and miR-320d respectively.

## References

[1] Bray F Ferlay J Soerjomataram I Siegel RL Torre LA Jemal A. Global Cancer Statistics 2018: GLOBOCAN Estimates of Incidence and Mortality Worldwide for 36 Cancers in 185 Countries. CA Cancer J Clin (2018) 68(6):394–424. doi: 10.3322/caac.21492 30207593

[2] Lheureux S Braunstein M Oza AM. Epithelial Ovarian Cancer: Evolution of Management in the Era of Precision Medicine. CA Cancer J Clin (2019) 69(4):280–304. doi: 10.3322/caac.21559 31099893

[3] Pujade-Lauraine E Banerjee S Pignata S. Management of Platinum-Resistant, Relapsed Epithelial Ovarian Cancer and New Drug Perspectives. J Clin Oncol (2019) 37(27):2437–48. doi: 10.1200/jco.19.00194 31403868

[4] Sölétormos G Duffy MJ Othman Abu Hassan S Verheijen RH Tholander B Bast RC Jr.. Clinical Use of Cancer Biomarkers in Epithelial Ovarian Cancer: Updated Guidelines From the European Group on Tumor Markers. Int J Gynecol Cancer (2016) 26(1):43–51. doi: 10.1097/igc.0000000000000586 26588231 PMC4679342

[5] Panni S Lovering RC Porras P Orchard S. Non-Coding RNA Regulatory Networks. Biochim Biophys Acta Gene Regul Mech (2020) 1863 194417(6):194417. doi: 10.1016/j.bbagrm.2019.194417 31493559

[6] Wang L Cho KB Li Y Tao G Xie Z Guo B. (lncRNA)-Mediated Competing Endogenous RNA Networks Provide Novel Potential Biomarkers and Therapeutic Targets for Colorectal Cancer. Int J Mol Sci (2019) 20(22):5758. doi: 10.3390/ijms20225758 PMC6888455 31744051

[7] Bi F An Y Sun T You Y Yang Q. PHGDH Is Upregulated at Translational Level and Implicated in Platin-Resistant in Ovarian Cancer Cells. Front Oncol (2021) 11:643129. doi: 10.3389/fonc.2021.643129 34178629 PMC8222667

[8] Lu TX Rothenberg ME. MicroRNA. J Allergy Clin Immunol (2018) 141(4):1202–07. doi: 10.1016/j.jaci.2017.08.034 PMC5889965 29074454

[9] Liang Y Zou Q Yu W. Steering Against Wind: A New Network of NamiRNAs and Enhancers. Genomics Proteomics Bioinf (2017) 15(5):331–37. doi: 10.1016/j.gpb.2017.05.001 PMC5673672 28882787

[10] Qi X Zhang DH Wu N Xiao JH Wang X Ma W. ceRNA in Cancer: Possible Functions and Clinical Implications. J Med Genet (2015) 52(10):710–8. doi: 10.1136/jmedgenet-2015-103334 26358722

[11] Ali Syeda Z Langden SSS Munkhzul C Lee M Song SJ. Regulatory Mechanism of MicroRNA Expression in Cancer. Int J Mol Sci (2020) 21(5):1723. doi: 10.3390/ijms21051723 PMC7084905 32138313

[12] Du B Wang T Yang X Wang J Shi X Wang X. SOX9, miR-495, miR-590-3p, and miR-320d Were Identified as Chemoradiotherapy-Sensitive Genes and miRNAs in Colorectal Cancer Patients Based on a Microarray Dataset. Neoplasma (2019) 66(1):8–19. doi: 10.4149/neo_2018_170324N214 30509082

[13] Hayes J Peruzzi PP Lawler S. MicroRNAs in Cancer: Biomarkers, Functions and Therapy. Trends Mol Med (2014) 20(8):460–9. doi: 10.1016/j.molmed.2014.06.005 25027972

[14] Choi YE Meghani K Brault ME Leclerc L He YJ Day TA. Platinum and PARP Inhibitor Resistance Due to Overexpression of MicroRNA-622 in BRCA1-Mutant Ovarian Cancer. Cell Rep (2016) 14(3):429–39. doi: 10.1016/j.celrep.2015.12.046 PMC4731274 26774475

[15] Belur Nagaraj A Knarr M Sekhar S Connor RS Joseph P Kovalenko O. The miR-181a-SFRP4 Axis Regulates Wnt Activation to Drive Stemness and Platinum Resistance in Ovarian Cancer. Cancer Res (2021) 81(8):2044–55. doi: 10.1158/0008-5472.Can-20-2041 PMC8137569 33574092

[16] Zou X Zhao Y Liang X Wang H Zhu Y Shao Q. Double Insurance for OC: miRNA-Mediated Platinum Resistance and Immune Escape. Front Immunol (2021) 12:641937. doi: 10.3389/fimmu.2021.641937 33868274 PMC8047328

[17] Muñoz-Galván S Felipe-Abrio B Verdugo-Sivianes EM Perez M Jiménez-García MP Suarez-Martinez E. Downregulation of MYPT1 Increases Tumor Resistance in Ovarian Cancer by Targeting the Hippo Pathway and Increasing the Stemness. Mol Cancer (2020) 19(1):7. doi: 10.1186/s12943-020-1130-z 31926547 PMC6954568

[18] Wang T Hao D Yang S Ma J Yang W Zhu Y. miR-211 Facilitates Platinum Chemosensitivity by Blocking the DNA Damage Response (DDR) in Ovarian Cancer. Cell Death Dis (2019) 10(7):495. doi: 10.1038/s41419-019-1715-x 31235732 PMC6591289

[19] Biamonte F Santamaria G Sacco A Perrone FM Di Cello A Battaglia AM. MicroRNA Let-7g Acts as Tumor Suppressor and Predictive Biomarker for Chemoresistance in Human Epithelial Ovarian Cancer. Sci Rep (2019) 9(1):5668. doi: 10.1038/s41598-019-42221-x 30952937 PMC6450929

[20] Sassu CM Palaia I Boccia SM Caruso G Perniola G Tomao F. Role of Circulating Biomarkers in Platinum-Resistant Ovarian Cancer. Int J Mol Sci (2021) 22(24):13650. doi: 10.3390/ijms222413650 34948446 PMC8707281

[21] Stuart GC Kitchener H Bacon M duBois A Friedlander M Ledermann J. 2010 Gynecologic Cancer InterGroup (GCIG) Consensus Statement on Clinical Trials in Ovarian Cancer: Report From the Fourth Ovarian Cancer Consensus Conference. Int J Gynecol Cancer (2011) 21(4):750–5. doi: 10.1097/IGC.0b013e31821b2568 21543936

[22] Tassi RA Gambino A Ardighieri L Bignotti E Todeschini P Romani C. FXYD5 (Dysadherin) Upregulation Predicts Shorter Survival and Reveals Platinum Resistance in High-Grade Serous Ovarian Cancer Patients. Br J Cancer (2019) 121(7):584–92. doi: 10.1038/s41416-019-0553-z PMC6889357 31434988

[23] Carvalho BS Irizarry RA. A Framework for Oligonucleotide Microarray Preprocessing. Bioinformatics (2010) 26(19):2363–7. doi: 10.1093/bioinformatics/btq431 PMC2944196 20688976

[24] Ritchie ME Phipson B Wu D Hu Y Law CW Shi W. Limma Powers Differential Expression Analyses for RNA-Sequencing and Microarray Studies. Nucleic Acids Res (2015) 43(7):e47. doi: 10.1093/nar/gkv007 25605792 PMC4402510

[25] Li JH Liu S Zhou H Qu LH Yang JH. Starbase V2.0: Decoding miRNA-ceRNA, miRNA-ncRNA and Protein-RNA Interaction Networks From Large-Scale CLIP-Seq Data. Nucleic Acids Res (2014) 42(Database issue):D92–7. doi: 10.1093/nar/gkt1248 PMC3964941 24297251

[26] Dweep H Sticht C Pandey P Gretz N. Mirwalk–Database: Prediction of Possible miRNA Binding Sites by “Walking” the Genes of Three Genomes. J BioMed Inform (2011) 44(5):839–47. doi: 10.1016/j.jbi.2011.05.002 21605702

[27] Yu G Wang LG Han Y He QY. Clusterprofiler: An R Package for Comparing Biological Themes Among Gene Clusters. Omics (2012) 16(5):284–7. doi: 10.1089/omi.2011.0118 PMC3339379 22455463

[28] Tamang S Acharya V Roy D Sharma R Aryaa A Sharma U. SNHG12: An LncRNA as a Potential Therapeutic Target and Biomarker for Human Cancer. Front Oncol (2019) 9:901. doi: 10.3389/fonc.2019.00901 31620362 PMC6759952

[29] Lu C Wei Y Wang X Zhang Z Yin J Li W. DNA-Methylation-Mediated Activating of lncRNA SNHG12 Promotes Temozolomide Resistance in Glioblastoma. Mol Cancer (2020) 19(1):28. doi: 10.1186/s12943-020-1137-5 32039732 PMC7011291

[30] Zhou B Li L Li Y Sun H Zeng C. Long Noncoding RNA SNHG12 Mediates Doxorubicin Resistance of Osteosarcoma *via* miR-320a/MCL1 Axis. BioMed Pharmacother (2018) 106:850–57. doi: 10.1016/j.biopha.2018.07.003 30119255

[31] Wang H Li H Zhang L Yang D. Overexpression of MEG3 Sensitizes Colorectal Cancer Cells to Oxaliplatin Through Regulation of miR-141/PDCD4 Axis. BioMed Pharmacother (2018) 106:1607–15. doi: 10.1016/j.biopha.2018.07.131 30119236

[32] Wang P Chen D Ma H Li Y. LncRNA MEG3 Enhances Cisplatin Sensitivity in Non-Small Cell Lung Cancer by Regulating miR-21-5p/SOX7 Axis. Onco Targets Ther (2017) 10:5137–49. doi: 10.2147/ott.S146423 PMC5661845 29123412

[33] Liu Y Yue P Zhou T Zhang F Wang H Chen X. LncRNA MEG3 Enhances (131)I Sensitivity in Thyroid Carcinoma *via* Sponging miR-182. BioMed Pharmacother (2018) 105:1232–39. doi: 10.1016/j.biopha.2018.06.087 30021359

[34] Yu Y Kou D Liu B Huang Y Li S Qi Y. LncRNA MEG3 Contributes to Drug Resistance in Acute Myeloid Leukemia by Positively Regulating ALG9 Through Sponging miR-155. Int J Lab Hematol (2020) 42(4):464–72. doi: 10.1111/ijlh.13225 32359033

[35] Huang H Chen J Ding CM Jin X Jia ZM Peng J. LncRNA NR2F1-AS1 Regulates Hepatocellular Carcinoma Oxaliplatin Resistance by Targeting ABCC1 *via* miR-363. J Cell Mol Med (2018) 22(6):3238–45. doi: 10.1111/jcmm.13605 PMC5980138 29602203

[36] Ke J Ni K Xue H Li J. RBM38 is Negatively Regulated by miR-320b and Enhances Adriamycin Resistance in Breast Cancer Cells. Oncol Lett (2022) 23(1):27. doi: 10.3892/ol.2021.13145 34868364 PMC8630814

[37] Wang J Zhao H Yu J Xu X Jing H Li N. MiR-320b/RAD21 Axis Affects Hepatocellular Carcinoma Radiosensitivity to Ionizing Radiation Treatment Through DNA Damage Repair Signaling. Cancer Sci (2021) 112(2):575–88. doi: 10.1111/cas.14751 PMC7894001 33251678

[38] Guo X Luo Z Xia T Wu L Shi Y Li Y. Identification of miRNA Signature Associated With BMP2 and Chemosensitivity of TMZ in Glioblastoma Stem-Like Cells. Genes Dis (2020) 7(3):424–39. doi: 10.1016/j.gendis.2019.09.002 PMC7452549 32884997

[39] Tomao F D’Incalci M Biagioli E Peccatori FA Colombo N. Restoring Platinum Sensitivity in Recurrent Ovarian Cancer by Extending the Platinum-Free Interval: Myth or Reality? Cancer (2017) 123(18):3450–59. doi: 10.1002/cncr.30830 28678350

[40] Colombo N Tomao F Benedetti Panici P Nicoletto MO Tognon G Bologna A. Randomized Phase II Trial of Weekly Paclitaxel vs. Cediranib-Olaparib (Continuous or Intermittent Schedule) in Platinum-Resistant High-Grade Epithelial Ovarian Cancer. Gynecol Oncol (2022) 164(3):505–13. doi: 10.1016/j.ygyno.2022.01.015 35063281

[41] da Costa A Baiocchi G. Genomic Profiling of Platinum-Resistant Ovarian Cancer: The Road Into Druggable Targets. Semin Cancer Biol (2021) 77:29–41. doi: 10.1016/j.semcancer.2020.10.016 33161141

[42] Xiao Y Lin FT Lin WC. ACTL6A Promotes Repair of Cisplatin-Induced DNA Damage, a New Mechanism of Platinum Resistance in Cancer. Proc Natl Acad Sci USA (2021) 118(3):e2015808118. doi: 10.1073/pnas.2015808118 33408251 PMC7826354

[43] Ma J Dong C Ji C. MicroRNA and Drug Resistance. Cancer Gene Ther (2010) 17(8):523–31. doi: 10.1038/cgt.2010.18 20467450

[44] Qi X Yu C Wang Y Lin Y Shen B. Network Vulnerability-Based and Knowledge-Guided Identification of microRNA Biomarkers Indicating Platinum Resistance in High-Grade Serous Ovarian Cancer. Clin Transl Med (2019) 8(1):28. doi: 10.1186/s40169-019-0245-6 31664600 PMC6820656

