# Supplementary figures and images for: MiR-320b and miR-320d as Biomarkers to Predict and Participate in the Formation of Platinum Resistance in Ovarian Cancer Patients
Source: Front Oncol. 2022 May 3;12:881496. doi: 10.3389/fonc.2022.881496 (PMC9110861; doi:10.3389/fonc.2022.881496)

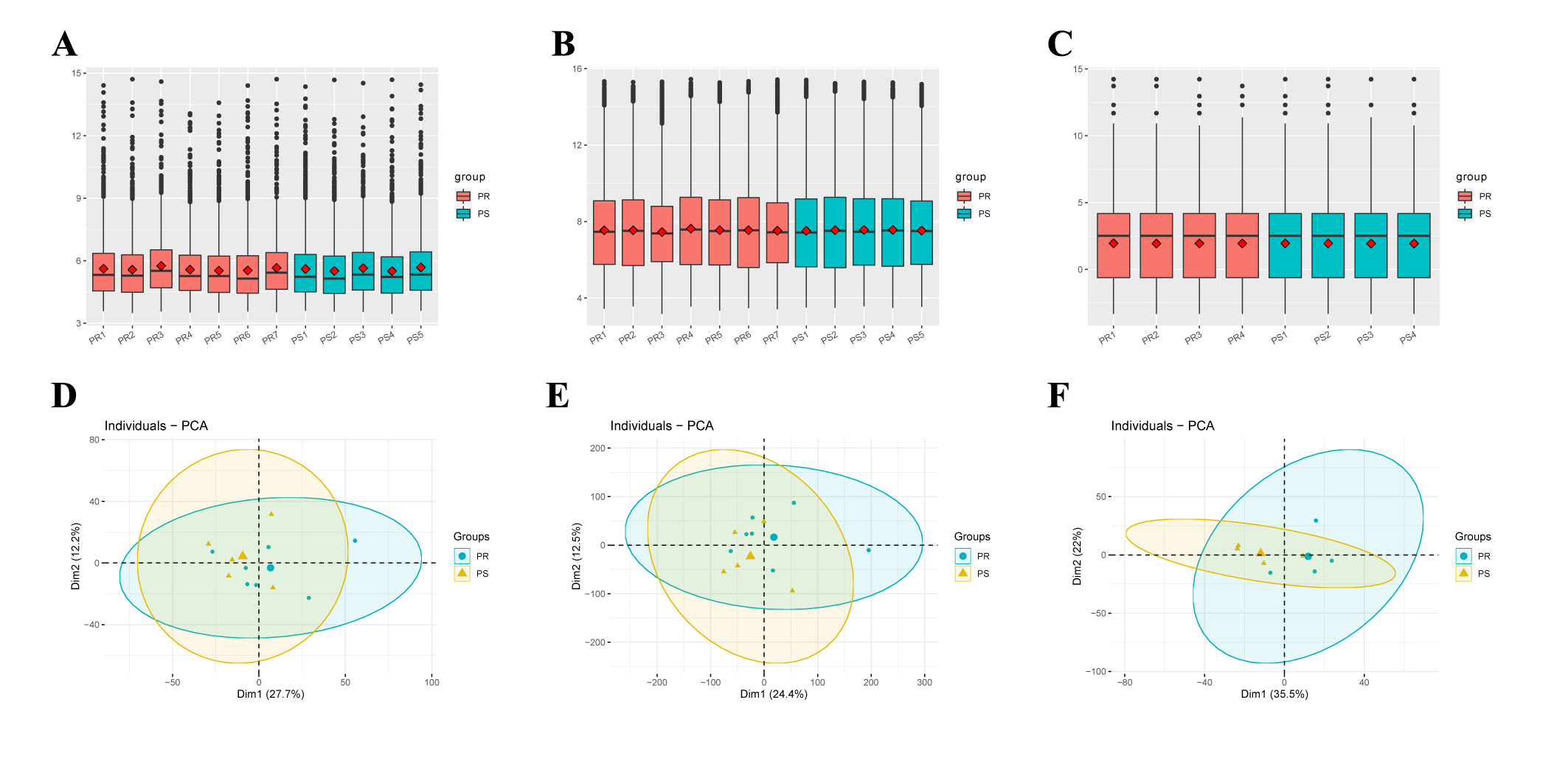

Supplement: Supplementary Figure 1 — Boxplot and PCA of differentially expressed lncRNAs, mRNAs and microRNAs. (A-C) Boxplot of differentially expressed lncRNAs, mRNAs and microRNAs between platinum-resistant and platinum-sensitive groups, respectively. (D-F) PCA of differentially expressed lncRNAs, mRNAs and microRNAs between platinum-resistant and platinum-sensitive groups, respectively. PCA, Principal Component Analysis; PR, platinum-resistant; PS, platinum-sensitive. [file Image_1.tif]

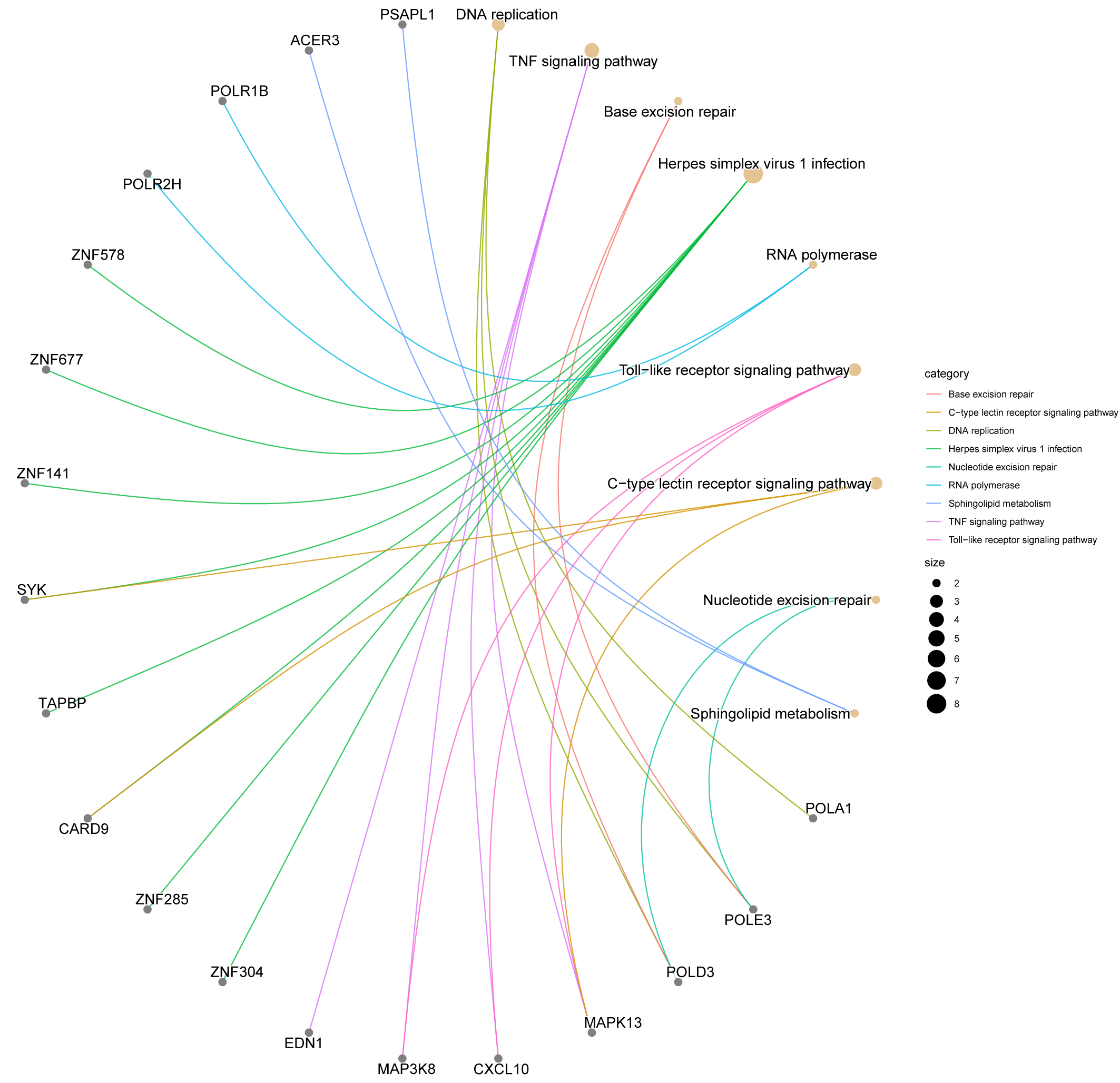

Supplement: Supplementary Figure 2 — Chord diagram of mRNAs enriched in KEGG pathways. Different colors on the right of the chord diagram represent different KEGG pathways, corresponding to legend, the left of the chord diagram represents mRNAs enriched in different KEGG pathways, and the line in the middle represents mRNAs are enriched in KEGG pathways. [file Image_2.tif]

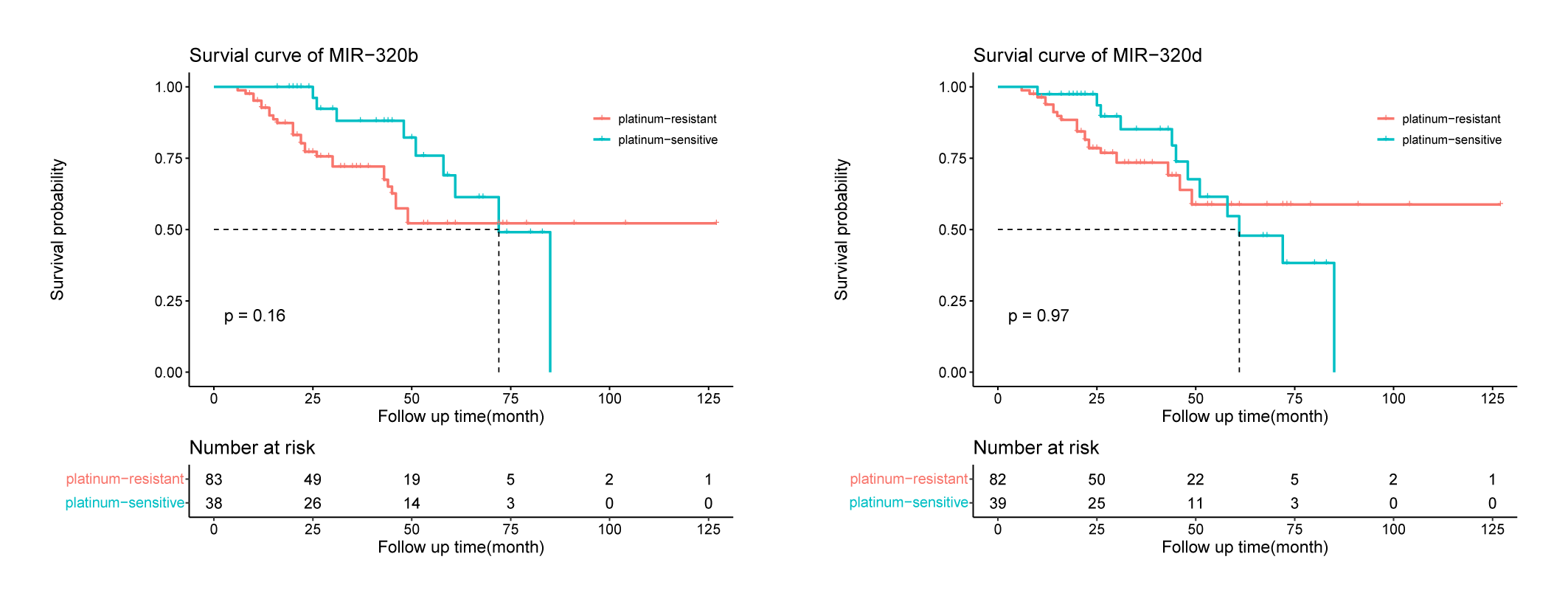

Supplement: Supplementary Figure 3 — Effect of miR-320b and miR-320d as biomarkers to predict overall survival in ovarian cancer patients receiving platinum-based chemotherapy by survival curves. (A, B) Survival curves of high expression group and low expression group judged by expression levels of miR-320b and miR-320d respectively. [file Image_3.tif]
